# Timber! Felling the loblolly pine genome

**DOI:** 10.1186/gb4170

**Published:** 2014-03-31

**Authors:** John P Hamilton, C Robin Buell

**Affiliations:** 1 Department of Plant Biology, Michigan State University, East Lansing, MI 48824, USA

## Abstract

Conventional short read sequences derived from haploid DNA were extended into long super-reads enabling assembly of the massive 22 Gbp loblolly pine, *Pinus taeda*, genome.

See related research http://genomebiology.com/2014/15/3/R59

## Text

The first plant genome sequenced was that of *Arabidopsis thaliana*, a homozygous inbred diploid, which had been selected specifically for its relatively small size (119 Mb assembly), limited repetitive sequence content, and homozygosity. However, not all species in the plant kingdom have simplistic genomes like *Arabidopsis*. Indeed, for the vascular land plants, the mean C value for angiosperms is 5.79 pg (5,662 Mbp), while that for gymnosperms is 18.08 pg (17,682 Mbp). These large genome sizes are attributable to amplification of repetitive sequences, whole or segmental genome duplication, and/or polyploidy - all of which pose a technical challenge in genome assembly with current short-read assembly approaches. In addition, many species are self-incompatible and/or outcrossing in nature, which can result in a high degree of heterozygosity that further confounds genome assembly. An article in this issue of *Genome Biology* overcomes many of these technical challenges to achieve a near complete assembly of the 22 Gbp loblolly pine genome [1].

## Reducing genome complexity by exploiting biology

**Figure 1:**
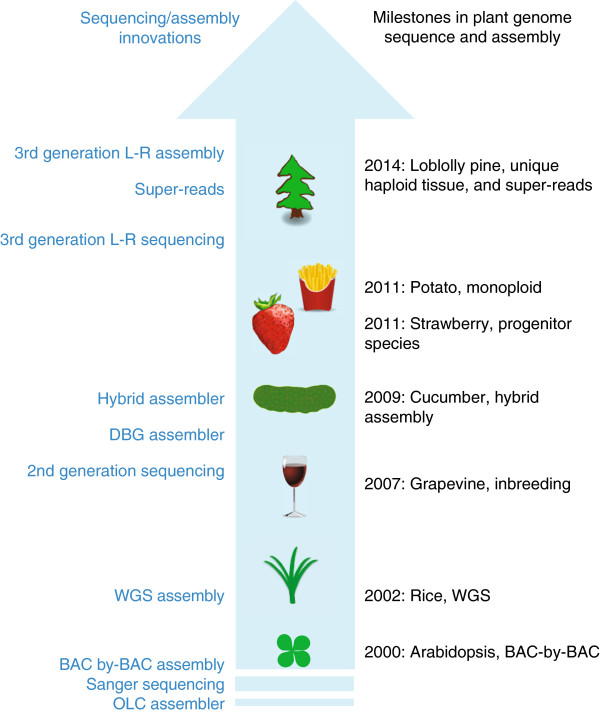
**Major innovations in sequencing and assembly, coupled with reductions in genome complexity, have led to major milestones in sequencing plant genomes, as highlighted by the loblolly pine genome.** Highlighted in the blue arrow are selected plants in which technical innovations in sequencing technology, genome assembly and/or a reduction in genome complexity were instrumental in achieving an assembled genome. Shown on the left (in blue) are major innovations in overall sequencing technologies, approaches and assembly starting with pre-2000 through to 2014 (OLC, overlap layout consensus; BAC, bacterial artificial chromosome; WGS, whole-genome shotgun sequencing; 2nd gen sequencing, second-generation sequencing; DBG, de Bruijn graph; 3rd gen L-R sequencing, third-generation long-read sequencing; 3rd gen L-R assembly, third-generation long-read assembly). Shown on the right (in black) are selected plants for which a milestone was achieved*: Arabidopsis thaliana* (119 Mbp assembly, 2000), *Oryza sativa* (rice, 390 Mbp assembly, 2002), *Vitis vinifera* (grapevine, 487.1 Mbp assembly, 2007), *Cucumis sativus* (cucumber, 243.5 Mbp assembly, 2009) *Fragaria vesca* (strawberry, 220 Mbp assembly, 2011), *Solanum tuberosum* (potato, 727 Mbp assembly, 2011), and *Pinus taeda* (loblolly pine, 22 Gbp assembly, 2014).

To reduce the complexity of plant genomes, researchers have been able to capitalize on genetics and/or reproduction to simplify the sequencing and assembly of a reference genome sequence (Figure 1). For example, heterozygosity can be reduced by 50% with each generation of inbreeding, as was shown for the grapevine genome [2]. For self-incompatible species that cannot be inbred, haploids or doubled haploids can be generated through crosses with a haploid inducer line, ovary/anther/microspore culture, or genome elimination (for review see [3]). Pollen and ovules are haploid, and future improvements in single-cell sequencing will facilitate whole-genome shotgun sequencing (WGS) of a wide range of plant taxa as the heterozygosity present in diploids could be bypassed. Polyploidy is common in the angiosperms, as exemplified by triploid banana, tetraploid potato and hexaploid bread wheat, and diploid progenitor species can be sequenced to bypass ploidy barriers. Cultivated strawberry is an octoploid, and researchers sequenced the diploid woodland strawberry *Fragaria vesca*[4] with a genome size of 240 Mbp, thereby avoiding the challenges in assembly of the larger and polyploid cultivated strawberry genome. However, while inherent knowledge of genetics and biology can be exploited to reduce the complexity of a plant genome, even a haploid genome of a typical gymnosperm is massive, and the generation of a robust genome assembly would be challenging at best.

The article in this issue of *Genome Biology* by Neale *et al.*[1], and two companion papers [5,6], is an excellent example of how a clever yet simple computational approach, coupled with the availability of sufficient haploid genomic DNA, has enabled the assembly of the 22 Gbp heterozygous loblolly pine (*Pinus taeda*) genome. In conifer seeds such as those of the loblolly pine, the haploid maternally derived megagametophye surrounds the developing embryo, providing it with nutrients, and is analogous to the endosperm in maize kernels. Due to the relatively large size of the megagametophyte, sufficient DNA for 11 paired-end libraries was isolated from a single, haploid loblolly pine megagametophyte to bypass the heterozygosity present in diploid tissues, such as needles. Using a novel computational approach described below, the authors complemented these haploid-derived libraries with 48 mate-paired and 9 fosmid ditag libraries constructed from ample DNA isolated from diploid needles, and constructed an assembly that spans 23.2 Gbp (20.1 Gbp non-gapped sequence), with an impressive N50 scaffold size of 66.9 Kbp. A total of 50,172 genes were annotated in the assembly and the quality of their assembly was evident in the detection of 201 of the 248 core CEGMA [7] genes, of which 91% were full-length. Interesting features of the loblolly pine genome included a high percentage of transposable elements (79% of the assembly), detection of extremely large introns (the largest was 318 Kbp) and 1,551 genes unique to conifers, of which 154 were absent in two related conifer species, *Picea abies* and *Picea sitchensis,* and restricted to loblolly pine [4].

## Innovations in genome assembly parallel innovations in sequencing technologies

The first plant genomes were sequenced using Sanger sequencing and initially utilized bacterial artificial chromosome (BAC)-by-BAC approaches, which later evolved into WGS. Assembly of the BACs and genomes was achieved using the overlap layout consensus (OLC) approach where all-versus-all pairwise read overlaps are identified, the read layout is calculated, and a contig consensus sequence is generated. The OLC assembly approach works well for high-quality long reads generated through Sanger sequencing. When ultra high-throughput next generation sequencing-by-ligation (SBL) and sequencing-by-synthesis (SBS) technologies emerged, a burst of innovation in genome assembly software occurred. The shorter read lengths, higher error rates and increased sequencing depth were incompatible with OLC assemblers due to very high memory usage and long assembly run times. A number of assemblers were developed based on the de Bruijn graph, an approach initially used for short reads generated by sequencing-by-hybridization and resurrected to assemble SBL and SBS reads efficiently. In the last few years, numerous improvements in short-read assembly have been made, including error correction of reads, trimming and filtering of low quality reads, merging of overlapping paired-end reads, scaffolding of contigs using large insert mate-pairs or RNA-sequencing reads, incorporating long reads, and gap filling using paired-end reads.

## Computer scientists to the rescue: super-reads

For large and complex genomes, the computational requirements of de Bruijn graph-based short-read assemblers can exceed what is available, even on large memory multi-core servers. Approaches such as parallel assemblers that distribute the memory and computational requirements across a cluster of interconnected nodes are one way to address this issue. Other options include assemblers optimized for low memory usage or assemblers that run in a specialized cloud-based distributed computing environment. However, more innovation in genome assembly and/or reduction in genome complexity will be needed to access large, complex genomes using current short-read sequences. One such strategy is that developed for the loblolly pine genome, which introduces a novel approach for assembling large complex genomes [4,5]. The core innovation is the reduction of approximately 15 billion error-corrected WGS reads (with an average length of 160 nucleotides) derived from haploid megagametophye tissue into approximately 150 million longer super-reads (with an average length of 362 nucleotides) using the MaSuRCA assembler [8]. The super-reads represent a 27-fold reduction in raw sequence and 100-fold reduction in number of paired-end reads. The long, high-quality super-reads could then be assembled with an OLC assembler and scaffolded using short-read mate-pairs and fosmid ditag libraries derived from diploid genomic DNA, thereby generating a higher quality assembly. The hybrid approach of generating super-reads then assembling them using an OLC assembler leverages the cost efficiencies of short-read sequencing platforms and leaves the door open to utilizing reads from current and future long-read sequencing technologies.

## Conclusions

With regard to biology, genome sequencing technology improvements that began in the late 1990s have continued through the present day, igniting research across a wide range of biological disciplines and creating entirely new fields of research, including genome biology and personal genomic medicine. None of this would have been possible without parallel improvements in algorithms, computer-processing capabilities, and applied computing solutions. Current long-read sequencing technologies such as Pacific Biosciences SMRT Sequencing [9] and Illumina TruSeq synthetic Long-Read Sequencing [10] are comparatively expensive for large genomes and are used mainly for scaffolding genomes and resolving haplotypes. Going forward, the cost for long-read sequencing will decrease and improvements in genome assembly software will leverage these long reads to generate higher quality and more complete complex genome assemblies at a reduced cost. Until then, the approach used by Neale *et al.* to sequence and assemble the loblolly pine genome provides a method of generating a high-quality draft assembly of a complex 22 Gbp plant genome using current and cost-efficient sequencing platforms.

## Abbreviations

BAC: Bacterial artificial chromosome; OLC: Overlap layout consensus; SBL: Sequencing-by-ligation; SBS: Sequencing-by-synthesis; WGS: Whole genome shotgun sequencing.

## Competing interests

The authors declare that they have no competing interests.
